# The extracellular proteome of *Rhizobium etli *CE3 in exponential and stationary growth phase

**DOI:** 10.1186/1477-5956-8-51

**Published:** 2010-10-14

**Authors:** Niurka Meneses, Guillermo Mendoza-Hernández, Sergio Encarnación

**Affiliations:** 1 Programa de Genómica Funcional de Procariotes, Centro de Ciencias Genómicas, Universidad Nacional Autónoma de México, Apdo. Postal 565-A, Cuernavaca, Morelos, CP 62210, México; 2 Departamento de Bioquímica, Facultad de Medicina, Universidad Nacional Autónoma de México, México, D.F., México

## Abstract

**Background:**

The extracellular proteome or secretome of symbiotic bacteria like *Rhizobium etli *is presumed to be a key element of their infection strategy and survival. Rhizobia infect the roots of leguminous plants and establish a mutually beneficial symbiosis. To find out the possible role of secreted proteins we analyzed the extracellular proteome of *R. etli *CE3 in the exponential and stationary growth phases in minimal medium, supplemented with succinate-ammonium.

**Results:**

The extracellular proteins were obtained by phenol extraction and identified by LC-ESI MS/MS. We identified 192 and 191 proteins for the exponential and stationary phases respectively. Using the software Signal P, we predicted signal peptides for 12.95% and 35.60% of the proteins identified in the exponential and stationary phases, respectively, which could therefore be secreted by the Sec pathway. For the exponential growth phase, we found in abundance proteins like the ribosomal proteins, toxins and proteins belonging to the group "defence mechanisms". For the stationary growth phase, we found that the most abundant proteins were those with unknown function, and in many of these we identified characteristic domains of proteases and peptidases.

**Conclusions:**

Our study provided the first dataset of the secretome of *R. etli *and its modifications, which may lead to novel insights into the adaptive response of different stages of growth. In addition, we found a high number of proteins with unknown function; these proteins could be analyzed in future research to elucidate their role in the extracellular proteome of *R. etli*.

## Background

Nitrogen-fixing symbioses between plants belonging to the family *Leguminosae *and soil bacteria collectively called rhizobia, contribute substantially to plant productivity. *Rhizobium etli *is a Gram-negative soil bacterium that carries out symbiosis with bean plants, specifically with *Phaseolus vulgaris*. The plant offers the bacteria a source of carbon (product of photosynthesis) in exchange for fixed nitrogen. This association allows legumes, like bean, to grow in nitrogen poor soil. To establish symbiosis the two partners exchange a series of molecular signals. In general, symbiotic or pathogenic processes are mediated by elicitors that are perceived by receptors on the cell membrane of the plant. These elicitors may be directly synthesized by bacteria and included in cell wall components, like lipopolysaccharides, proteins or peptides, or they may be low molecular weight compounds released from the plant cell wall by bacterial enzymes during the infection process [1]. These effectors are commonly referred to as microbe-associated molecular patterns (MAMPs). MAMPs are mainly surface-associated molecules that mostly cannot be readily altered and are typical of classes of microorganisms [2]. They are usually related with virulence factors and another group of secreted proteins. These are usually degrading enzymes that are secreted actively and support the early and late stages of infection [1].

The secreted proteins are very important in the adaptation of the bacteria to the environment, as well as in the interaction with other organisms. These proteins have different functional roles such as degradation of substrates as well as cell wall turnover and sensing [3]. However, the role, of many of the proteins secreted by *R. etli *is unknown.

In this study, we used a proteomic approach in order to display the extracellular proteome (secretome) of *R. etli *CE3 cultivated in minimal medium.

The extracellular proteins were extracted from the culture supernatant of *R. etli *CE3 in exponential and stationary growth phases. These extracts were analyzed by LC-ESI MS/MS, which allowed us to classify the proteins identified in functional groups. We also carried out, a bioinformatics analysis to identify proteins having signal peptides. The collected data from the extracellular proteome of *R. etli *CE3 cultivated in minimal medium, should give an insight into which proteins are secreted during two of the most important phases of growth (exponential and stationary phases), and how these proteins could participate in the survival of the bacteria in its environment and during the interaction between *R. etli *and its host plant (*P. vulgaris)*.

## Materials and methods

### Cell culture and preparation of culture supernatant

*Rhizobium etli *strain CE3 was grown as previously described [4], at 30°C in 2 L of minimal medium (0.2 mM magnesium sulphate; 0.1 mM potassium monoacid phosphate; 0.2 mM succinic acid, 0.1 mM ammonium chloride, 1 mM calcium chloride) for 6 h and 24 h for exponential and stationary phases, respectively. Cells were pelleted by centrifugation (7500 × g at 4°C, 30 min). The liquid phase was centrifuged for 45 min at 6500 × g at 4°C. The cell-free supernatant was frozen at 80°C and lyophilized to complete dryness.

### Extraction of extracellular proteins

The lyophilized supernatant was rehydrated in 5 ml extraction buffer (0.7 M sucrose; 0.5 M tris-base; 0.1 M potassium chloride; 50 mM EDTA; 2% β-mercaptoethanol; 12 mg/ml Polyvinylpolypyrrolidone PVPP; 30 mM hydrochloric acid), 5 ml water-saturated phenol was added, mixed thoroughly and incubated at RT for 45 min on a tilting table. The mixture was centrifuged at 3000 × g at 4°C for 30 min and the phenol phase was transferred to a fresh tube, supplemented with 200 μl of 1 M 1,4-dithiothreitol (DTT) and 300 μl of 8 M-ammonium acetate and incubated for 30 min. The phenol extraction was used to separate the proteins from the exopolysaccharides, DNA and other molecules that were also secreted in large amounts by *R. etli *CE3. Proteins were precipitated by the addition of 25 ml of -20°C methanol and incubation at -20°C for at least 12 h. Subsequently, proteins were pelleted by centrifugation (5000 × g at 4°C for 10 min). The precipitate was washed twice with 10 ml cold 80% (v/v) acetone, and dried at RT, and resuspended in 200 μl of solubilization buffer (7 M urea; 2% CHAPS; 1 mM DTT). For each experiment, we applied up to 500 μg of protein sample.

### Tryptic digest and RP/LC prefractionation

After adjusting the pH to 8.0 with 100 mM Tris-HCl, the samples were reduced by adding 1 mM dithiothreitol and alkylated in 10 mM iodoacetamide. Proteins were then precipitated with 10% trichloroacetic acid, washed twice with 95% acetone and resolubilized in 100 mM Tris-HCl, pH 8.0. Digestion was performed overnight at 37°C with porcine modified trypsin (1/50, w/w, trypsin/sample). In order to simplify the complexity of the peptide mixture, tryptic digests were subjected to an off-line reverse-phase LC prefractionation of peptides on an Aquapore OD-300, 2.1 × 100 mm C18 column (Perkin-Elmer Corporation, Norwalk, USA). Digests were acidified with trifluoroacetic acid (TFA) and loaded onto the column previously equilibrated in 95% buffer A (0.1% TFA in water), 5% buffer B (0.08% TFA in acetonitrile). The chromatographic separation was developed at a flow rate of 100 μl/min as follows: 5-15% buffer B over 10 min, 15-35% buffer B over 65 min, 35-80% buffer B over 15 min, and a 10 min hold at 80% buffer B. Fractions of 0.5 ml were collected, dried by centrifugal evaporation and dissolved in 20 μl of 1% acetic acid prior to analysis by on-line LC-ESI-MS/MS.

### LC-ESI-MS/MS

MS/MS analysis of each fraction obtained from the off-line separation step was carried out on a 3200 Q TRAP hybrid tandem mass spectrometer (Applied Biosystems/MDS Sciex, Concord, ON, Canada), equipped with a nano electro spray ion source (NanoSpray II). The instrument was coupled on-line to a nanoAcquity Ultra Performance LC system (Waters Corporations, Milforrd, MA). Samples were desalted by injection onto a Symmetry C18 UPLC trapping capillary column (180 μm × 20 mm, Waters Corporations) and washed with 0.1% formic acid in 100% MilliQ water at a flow rate of 15 μl/min. After 3 min, the trap column was switched in-line with the capillary analytical column. Peptides were separated on an ethylene bridged hybrid, C18 UPL column (75 μm × 100 mm, Waters Corporations) using a linear gradient of 2-70% acetonitrile, 0.1% formic acid over a 60 min period, at a flow rate of 0.25 μl/min. Spectra were acquired in automated mode using Information Dependent Acquisition (IDA). Precursor ions were selected in Q1 using the enhanced MS mode with a scan range of *m*/*z *400-1500 and 4000 amu/s. Selected ions were subjected to an enhanced resolution scan at the low speed of 250 amu/s over a narrow (30 amu) mass range and then to an enhanced product ion scan (MS/MS). The precursor ions were fragmented by collision activated dissociation (CAD) in the Q2 collision cell using rolling collision energy. The fragment ions generated were captured and mass analyzed in the Q3 linear IT.

### Bioinformatics analysis of the extracellular proteome of R. etli *CE3*

Data interpretation and protein identification were performed from the MS/MS spectra datasets using the MASCOT search algorithm (Matrix Science, London, UK available at http://www.matrixscience.com). Data were queried against a *R. etli *CE3 database (Retli_Marzo 2008, NC_007761.1). Trypsin was set as the specific digest reagent, the precursor mass tolerance and fragment ion tolerances equalled 0.5 and 0.8 Da, respectively, one missed cleavage site was allowed, and carbamidomethyl-cysteine as a fixed and methionine oxidation set as a variable modification. The protein identification reporting criteria included at least two MS/MS spectrum matched at the 95% level of confidence (Mowse score = 25 in the conditions used in this work) and the presence of a consecutive *y *and/or *b *ion series of three or more amino acids.

(Mowse score = -10×log_10_(p), where p is the likelihood that the identification is a random event).

### N-terminal signal-peptide prediction

The SignalP 2.0 software (http://www.cbs.dtu.dk/services/SignalP-2.0/) was used with the settings for Gram-negative organisms. Signal peptides were predicted by a Hidden Markov Model as well as by a neural networks method. Protein classification by cluster orthologous groups (COG) was performed on the NCBI server (http://www.ncbi.nih.gov/COG).

## Results and discussion

A fundamental biological goal in bacteriology is the identification of gene expression patterns for all major functional classes during the exponential and stationary phases, and to relate these changes to important physiological events. To complement this knowledge, the goal must be to determine the specific role of different set of proteins secreted in the different growth phases. Towards this goal, we performed secretome analysis of *R. etli *batch cultures during the exponential-growth phase and early stationary phase with the purpose of identifying the extracellular proteome in these phases of growth. To do this we cultured a *R. etli *in Minimal Medium (MM) succinate-ammonium, because in the rizosphere *R. etli *lives in conditions similar to those in minimal medium; consequently growing *R. etli *in minimal medium may give a more realistic idea about the behaviour of this bacterium on its environment. In addition, the fermentative state of free living *R. etli *in MM may be closely related to the metabolism that these bacteria express during the infection process and nitrogen fixation in symbiosis [4,5].

Previous studies showed that *R. etli *under fermentative growth conditions reached mid-exponential phase, and stationary phase at 6 h and 24 h respectively [4,5]. To reconfirm this, we cultured *R. etli *CE3 in MM, and demonstrated that our collection times represent both growth phases (data not show). Furthermore, the availability of nitrogen, succinate and oxygen, was measured throughout the incubation, to determine if some factors become limited and cause the culture to reach the stationary phase (data not shown), because any kind of restriction could alter protein synthesis, which would have an impact on the composition of the proteome. We concluded that the only factor limiting is oxygen, which becomes limiting after 10 h (this coincides with the culture entering pre-stationary phase). The other compounds (nitrogen and succinate) measured in the culture medium are in sufficient concentration in the period preceding sampling.

The extracellular proteins of *R. etli *CE3 of exponential or stationary growth phase were obtained from cell-free culture supernatant as indicated in Materials and Methods. A phenol extraction method was applied to separate the proteins from exopolysaccharides. Proteins derived from this purification method were analyzed by LC-MS/MS. Two independent experiments of each growth condition were run using 500 μg of protein.

**Figure 1:**
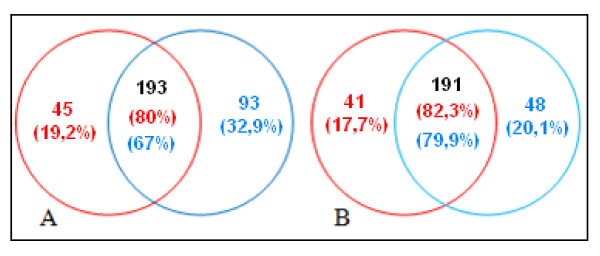
**Common proteins found between two experiments and the number of indentified proteins in each experiment**. Diagram A corresponds to the *R. etli *experiments in exponential phase. Diagram B corresponds to the *R*. *etli *experiments in stationary phase.

By the criteria specified in Materials and Methods, we identified 327 and 280 proteins for the exponential and stationary phase, respectively; and a total of 192 and 191 proteins were found reproducibly in both replicates for each growth phases (Figure 1). Being the percentage of reproducibility for each pair of trials; 67% and 80%, (exponential phase) 82.3% and 79.9% (stationary phase) (Figure 1). Information about the identified proteins is given in the Additional file 1 and Additional file 2.

The proteins identified were grouped by COG (Cluster Orthologus Groups), according to NCBI. The proteins could be allocated to nineteen functional groups, the largest groups being translation, ribosomal structure and biogenesis, carbohydrate transport and metabolism, post-translational modification, protein folding, chaperons and unknown protein function.

## Proteins secreted by *R. etli *CE3 in exponential phase

The proteins identified in the exponential growth phase were mainly proteins involved in bacterial physiology and survival. The most represented COGs were "translation, ribosomal structure and biogenesis" with 43 proteins identified, followed by the "energy production and conversion" that represented 19 proteins; "post-translational modification, folded proteins and chaperons" with 17 proteins; "metabolism and transport of carbohydrates" with 15 proteins; "amino acid transport and metabolism" 13 proteins; "cell wall, membrane, envelope biogenesis" with 10 proteins. The rest of the groups represented less than 5% of the total of proteins (Table 1).

**Table 1:** Comparison of the extracellular proteins of *R. etli*; identified in the exponential growth phase (6 h) and the stationary growth phase (24 h)

COGs	prot 6 h (%)	prot 6 h (SP) (%)	prot 24 h (%)	prot 24 h (SP) (%)
**Energy production and conversion (C)**	**9.8**	1.0	3.1	-

Cell cycle control, cell division, chromosome partitioning (D)	0.5	-	0.5	-

**Amino acid transport and metabolism (E)**	**6.7**	1.0	**7.3**	4.7

Nucleotide transport and metabolism (F)	1.0	-	-	-

**Carbohydrate transport and metabolism (G)**	**7.7**	1.0	**7.9**	4.1

Coenzyme transport and metabolism (H)	1.5	-	0.5	-

Lipid transport and metabolism (I)	1.0	-	0.5	-

**Translation, ribosomal structure and biogenesis (J)**	**22.3**	-	**6.3**	-

Transcription (K)	3.1	-	3.1	-

**Replication, recombination and repair (L)**	3.6	-	**5.2**	0.5

**Cell wall, membrane, envelope biogenesis (M)**	**5.1**	1.5	**7.3**	3.7

Cell motility (N)	3.6	1.5	2.0	0.5

**Posttranslational modification, protein turnover, chaperones (O)**	**8.8**	1.0	**6.8**	1.0

**Inorganic ion transport and metabolism (P)**	3.1	0.5	**5.7**	3.7

Secondary metabolites biosynthesis, transport and catabolism (Q)	2.6	0.5	0.5	-

General function prediction only (R)	2.0	-	3.6	1.6

**Function unknown (S)**	3.6	1.5	**28.8**	**11.0**

Signal transduction mechanisms (T)	3.1	1.0	1.0	-

Intracellular trafficking, secretion, and vesicular transport (U)	4.1	0.5	1.5	1.6

Defense mechanisms (V)	1.5	-	1.0	-

**Without COG (WC)**	4.1	1.5	**6.2**	3.1

Total protein	192	25	191	68

The proteins were grouped in functional groups (COGs). The relative contribution to each COG of proteins with a potential signal peptide (SP) is indicated. Functional groups with a major abundance in proteins are shown in bold letters and numbers.

From the identified proteins, those corresponding to defence mechanisms are; probable antibiotic resistance (streptomycin kinase) protein, putative 6-aminohexanoate-dimer hydrolase protein and probable efflux cation transporter protein (Additional file 1). These proteins are essential since they are involved in the bacteria defence against other organisms [6].

ThuK (trehalosemaltose ABC transporter) and RHE_CH03989 (Probable sugar ABC transporter, ATP-binding protein) are two ABC proteins identified in exponential phase predicted to be involved in carbohydrate transport, which contained a conserved ATP-binding Walker A region (GXXGXGKT/S) [7], and were probably secreted by the type I protein secretion systems.

In this growth phase were also identified ClpX, an ATP-dependent Clp protease and heat shock protein. These proteins were also identified on the cell surface of *Corynebacterium glutamicum*, which might play a role in the degradation of misfolded proteins, and assist the refolding of proteins related to stress conditions [8]. The presence of proteins DegPch2, a serine protease DO-like protein, and Lon protein, an ATP-dependent protease LA protein, in addition to their participation in stress response, could suggest a putative role in the degradation of plant defensive barriers.

In our study, proteins found in the extracellular proteome in exponential growth phase, are also identified in the cytoplasmic fraction like SucB, SdhA, AtpA, AtpC, AtpD and AtpH proteins (data not shown). These proteins were reported also on the cell surface of *C. glutamicum*, and were related to stress response [8].

In agreement with our data, Kazemi-Pour et al [9], found many *Erwinia chrysanthemum *cytoplasmic proteins in the secretome. These proteins, identified also by us in the secretome of *R. etli*, included two major cytoplasmic proteins, the elongation factor EF-Tu and the chaperonin GroEL, two cytoplasmic enzymes, AcnB and RhaA, and periplasmic proteins involved in chemotaxis (Tsr) and transport (OppA). These proteins perform functions within the cell such as protein synthesis and carbon metabolism. Derived from their function and supported by the results of the Signal P analysis (see below), it is most likely that these proteins are not actively exported into the culture medium. Cell lysis could release proteins into the extracellular space, contaminating the extracellular proteome with cytosolic proteins. To assess if cytosolic proteins originating from cell lysis and contaminated the secretome, we compared the protein profiles of whole cell lysates with those of extracellular proteins using two-dimensional gel electrophoresis (Additional file 3). The protein composition of extracellular proteins was considerably different from that of whole cell samples. Moreover, a direct relationship between the relative spot volume in the cellular proteome and secretome was not found, suggesting that very little cell lysis occurred during growth and subsequent procedures. However, although these data suggest that large scale lysis does not occur during cultivation, we cannot totally rule out that the cytosolic proteins in the extracellular space are proteolysis resistant remains of lysed cells. Cell lysis during harvesting and processing of the sample can be ruled out, because this should release all of the most abundant cytosolic proteins into the extracellular space, which should then be detected in the extracellular proteome.

Additionally, in this phase of growth, we unexpectedly identified a great number of proteins belonging to the translation, ribosomal structure and biogenesis group. Together with elongation factor EF-Tu protein (TufB) which was also identified by Kazemi-Pour [9], we found; 16, 30S ribosomal proteins, 19, 50S ribosomal protein, a polyribonucleotide nucleotidyltransferase protein (Pnp), aspartyl-tRNA synthetase protein (AspS), elongation factor G protein (FusA2), glutamyl-tRNA amidotransferase subunit B protein (GatB), alanyl-tRNA synthetase protein (AlaS2) and glutamyl-tRNA amidotransferase subunit A protein (GatA2) (Additional file 1).

When we compared the secreted proteins obtained during the exponential phase versus stationary phase, we observed that this group of proteins (detected in both experimental replicates of exponential phase growth) practically disappeared (Additional file 1 and 2), corroborating our conclusion that they are not mainly product of cell lysis. Therefore, cellular lysis during growth is not likely to be the sole reason for the appearance of cytosolic proteins in the extracellular proteome [1].

Chaperones and translation elongation factors were also found in secretomes of *Xanthomonas campestris *pv. *campestris *[10], *Corynebacterium efficiens *[11], *Bacillus anthracis *and *Bacillus thuringiensis*[12]. Our interpretation is that these proteins are involved in other cellular processes unrevealed until now, and these examples suggest that typical cytosolic proteins might have additional functions outside the cell.

Proteomic analysis of subcellular fractions of *Mycobacterium tuberculosis *revealed that some of the proteins, like Ef-Tu, could also be found in cell wall and membrane compartments [13]. In *Escherichia coli*, a similar result was reported [14] suggesting that these proteins are secreted through the outer membrane vesicles. Our preliminary results suggest that in *R. etli *CE3 similar export mechanisms are operating (data not shown), allowing the export of cytoplasmic, periplasmic and outer membrane proteins to the medium.

## Proteins secreted by *R.etli *CE3 during stationary phase

Among the 191 proteins identified in the extracellular proteome in the stationary phase growth (Additional file 2), 55 proteins were without similarity to functionally characterized proteins previously (COG s, "function unknown"), which represents 39.9% of the total of proteins identified in this growth phase. In addition, 21 of the proteins identified in this COG had predicted *N*- terminal signal peptide, showing the there are many proteins with unknown functions in the extracellular proteome of *R. etli *CE3.

Other overrepresented COGs were "metabolism and carbohydrate transportation" with 15 proteins; "metabolism and amino acid transport" with 14 proteins; "cellular wall, membrane, biogenesis" with 14 proteins; "post-translational modification, protein folding, chaperons" with 13 proteins; and "translation, ribosomal structure and biogenesis" with 12 proteins (Additional file 2).

Among the proteins found in the secretome in this phase of growth, we identified a polysaccharidase protein (PlyA2). This protein could be crucial in the defence of *R. etli*, or in root colonization as was reported in *Rhizobium leguminosarum*, where PlyA showed the capacity to degrade exopolysaccharide (EPS) and carboxymethyl cellulose (CMC) [15]. We also identified the VirE3 protein (Additional file 2). In *Agrobacterium tumefaciens*, it has been shown that VirE3 is related to the infection process [16]. Therefore, this protein in *R. etli *could be associated with the process of colonization of the plant.

Studies carried out with MAMP (microbe associated molecular patterns) and their perceptions by plants have been mainly performed with phytopathogenic bacteria [17]. These studies revealed that plants have developed perception systems for different bacterial MAMPs like flagellin, lipopolysaccharide (LPS), elongation factor Tu (EF-Tu), cold shock protein (CSP) or peptidoglycan (PGN) [17-20]. Several plant responses to MAMP molecules, occurring within minutes, include ion-flux across the plasma membrane, increased intracellular Ca^2+ ^concentration, oxidative burst, MAP kinase activation and major transcriptional changes [17]. Interestingly, many of these responses have also been detected in epidermal cells of legume roots soon after application of Nod Factors [21]. In this study, we identified FlaCch1, FlaCe, FlaCch2, FlaCch3 and elongation factor Tu proteins in the extracellular proteome of *R. etli *CE3, suggesting that *R. etli *could also use these proteins for the initial recognition between the bacteria and the plant, as was reported previously in other bacterial models [18]. The release of flagellar proteins into the extracellular medium is commonly observed in bacterial cultures, because the flagellum is easily disrupted from the cell surface, however, in *C. jejuni *these proteins are secreted by the flagellar system and mutants in the *flaC *gene showed a significantly reduced level of invasion into HEp-2 cells [22].

The large amount of proteins (55) with unknown function identified under stationary growth condition is remarkable. The analysis and study of those proteins will allow the discovery of new functions in the bacteria. We carried out a search for domains using bioinformatics tools [http://www.uniprot.org], and found that several of the proteins have domains that could predict important physiological functions in symbiosis. 1) Proteins with domains of CLE (CLAVATA3/ESR-related), a class of plant peptides known to play roles as short-range signals in controlling the fate of stem cells; 2) peptidases; 3) adhesion proteins; 4) PKD, a domain first identified in the Polycystic kidney disease protein; 5) RTX toxins (RTX representing repeats in the structural toxin) which are important virulence factors produced by a wide range of Gram-negative bacteria; among others (Table 2). In general, these domains belong to enzymes that could be used by the bacteria in defence and adaptation to the environment [23,24]. However, we need additional experiments to determine their possible role in the *R. etli *secretome.

**Table 2:** Functional domains present in some hypothetical proteins secreted by *R. etli* in stationary growth phase

ID from hypothetical proteins	Domain	Domain description found in Swissprot data base
gi|21492958	IPB000209	Proteolytic enzymes that exploit serine in their catalytic activity are ubiquitous, being found in viruses, bacteria and eukaryotes, (serine-carboxyl peptidases).

gi|86355844	IPR010662	The structure shows an alpha-beta hydrolase fold suggesting an enzymatic function for these proteins. The crystal structure from *B. subtilis *has been solved.

gi|86356671	PDOC00169	Cytochrome c-type centres are also found in the active sites of many enzymes, including cytochrome cd1-nitrite reductase as the *N*-terminal haem c domain, in quinoprotein alcohol dehydrogenase as the C-terminal domain, in Quinohemoprotein amine dehydrogenase A chain as domains 1 and 2, and in the cytochrome bc1 complex as the cytochrome bc1 domain.

gi|86357010	IPR010221 VCBS	**IPR006644 Dystroglycan-type cadherin-like** In animals, cadherin domain-containing proteins are adhesion molecules that modulate a wide variety of processes including cell polarization and migration but they have also been identified in yeast and magnetotactic bacteria. Crystal structures have revealed that multiple cadherin domains form Ca^2+^-dependent rod-like structures with a conserved Ca^2+^-binding pocket at the domain-domain interface. **IPR008009 Putative Ig** This domain probably corresponds to a new superfamily in the immunoglobulin fold. The function of this domain is uncertain it may be involved in cell adhesion. In the Sushi repeat-containing protein (SrpX), this domain is found between two sushi repeats. PKD domains are also found in other proteins, usually in the extracellular parts of proteins involved in interactions with other proteins. For example, domains with a PKD-type fold are found in archaeal surface layer proteins that protect the cell from extreme environments. **IPR005066 Moybdenum cofactor oxidoreductase, dimerisation** The majority of molybdenum-containing enzymes utilise a molybdenum cofactor (MoCF or Moco) consisting of a Mo atom coordinated via a cisdithiolene moiety to molybdopterin (MPT). MoCF is ubiquitous in nature, and the pathway for MoCF biosynthesis is conserved in all three domains of life. MoCF-containing enzymes function as oxidoreductases in carbon, nitrogen, and sulphur metabolism. This domain of about 100 residues is found multiple (up to 35) copies in long proteins from several species of Vibrio, Colwellia, Bradyrhizobium, and Shewanella (hence the name VCBS) and in smaller copy numbers in proteins from several other bacteria. The large protein size and repeat copy numbers, species distribution, and suggested activities oouter membrane adhesin like protein.

gi|86357471	EC:2	**IPR013785 Aldolase-type TIM barrel** This entry represents the TIM beta/alpha barrel found in aldolase and related proteins. This TIM barrel usually covers the entire protein structure. Proteins containing this TIM barrel domain include class I aldolases, class I DAHP synthases, class II fructose-bisphosphate aldolases (FBP aldolases), and 5-aminolaevulinate dehydratase (a hybrid of classes I and II aldolases) **IPR006638 Elongator protein 3/MiaB/NifB** This domain is found in MoaA, NifB, PqqE, coproporphyrinogen III oxidase, biotin synthase and MiaB families, and includes a representative in the eukaryotic elongator subunit, Elp-3. Some members of the family are methyltransferases **IPR007197 Radical SAM** Radical SAM proteins catalyze diverse reactions, including unusual methylations, isomerization, sulphur insertion, ring formation, anaerobic oxidation and protein radical formation. Evidence exists that these proteins generate a radical species by reductive cleavage of S:-adenosylmethionine (SAM) through an unusual Fe-S centre.

gi|86357955	EC:4.2.3.5	Chorismate synthase catalyzes the last of the seven steps in the shikimate pathway which is used in prokaryotes, fungi and plants for the biosynthesis of aromatic amino acids. It catalyzes the 1,4-trans elimination of the phosphate group from 5-enolpyruvylshikimate-3-phosphate (EPSP) to form chorismate which can then be used in phenylalanine, tyrosine or tryptophan biosynthesis. Chorismate synthase requires the presence of a reduced flavin mononucleotide (FMNH2 or FADH2) for its activity. Chorismate synthase from various sources shows a high degree of sequence conservation. It is a protein of about 360 to 400 amino-acid residues).

gi|86358739	GI:241205844	Integral membrane protein which is thought to regulate cation conductance. A variety of proteins belong to this family. These include the prohibitins, cytoplasmic anti-proliferative proteins and stomatin, an erythrocyte membrane protein. Bacterial HflC protein also belongs to this family.

gi|86360288	PS00495	The apple domain has an *N*-terminal region that contains four tandem repeats of about 90 amino acids and a C-terminal catalytic domain. The 90 amino-acid repeated domain contains 6 conserved cysteines. It has been shown that three disulphide bonds link the first and sixth, second and fifth, and third and fourth cysteines. This entry contains apple-like domains, which are presented in Plasminogen, *Caenorhabditis elegans *hypothetical ORFs and the extracellular portion of plant S-locus glycoproteins and S-receptor kinases. The domain is predicted to possess protein-and/or carbohydrate-binding functions.

gi|86360818	PF 06724	This region consists of two a pair of transmembrane helices and occurs three times in each of the family member proteins.

gi|89255303	PF04972	The BON domain is typically ~60 residues long and has an alpha/beta predicted fold. There is a conserved glycine residue and several hydrophobic regions. This pattern of conservation is more suggestive of a binding or structural function rather than a catalytic function. Most proteobacteria seem to possess one or two BON-containing proteins, typically of the OsmY-type proteins; outside of this group the distribution is more disparate.

## Comparison of the secretome of *R. etli *during exponential and stationary growth phases

**Figure 2:**
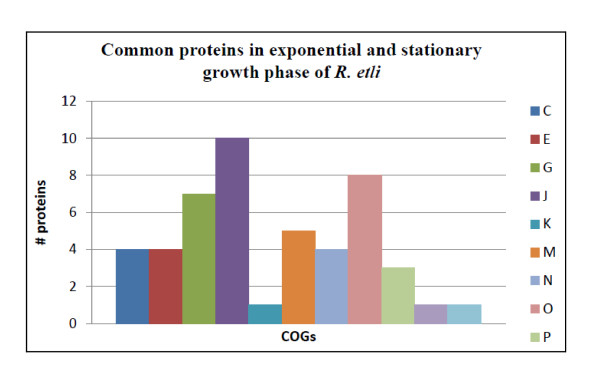
**Proteins identified in both growth phases**. Proteins were grouped into functional groups (COG). Energy production and conversion (C); Amino acid transport and metabolism (E); Carbohydrate transport and metabolism (G); Translation, ribosomal structure and biogenesis (J); Transcription (K); Cell wall, membrane, envelope biogenesis (M); Cell motility (N); Posttranslational modification, protein turnover, chaperones (O); Inorganic ion transport and metabolism (P); General function prediction only (R); Intracellular trafficking, secretion, and vesicular transport (U).

The composition of the secretome of *R. etli *CE3 growing in either exponential or stationary phase revealed some similarities. Forty eight proteins were identified as being secreted in both, growth conditions, (Figure 2 and Additional file 4). These proteins could be important components of the *R. etli *CE3 secretome and play essential roles in this bacterium since they are present in both growth phases.

In the exponential phase 144 proteins were found as specific components of the *R. etli *secretome with the most represented COGs being "energy production and conversion", "amino acid transport and metabolism", "translation, ribosomal structure and biogenesis", "posttranslational modification, protein turnover, chaperones" (Table 3). For the stationary phase 143 proteins were identified as specific, the most represented COGs being "amino acid transport and metabolism", "carbohydrate transport and metabolism" and "function unknown" (Table 3), which suggests that there are specific bacterial requirements in each growth phase.

**Table 3:** Comparison of specific proteins for each growth phase

COGs	prot 6 h (%)	prot 6 h (SP) (%)	prot24 h (%)	prot 24 h (SP) (%)
**Energy production and conversion (C)**	**10.3**	1.3	1.5	-

Cell cycle control, cell division, chromosome partitioning (D)	0.7	-	0.7	-

**Amino acid transport and metabolism (E)**	6.2	1.3	**7.2**	5.1

Nucleotide transport and metabolism (F)	1.3	-	-	-

Carbohydrate transport and metabolism (G)	**5.5**	-	5.8	5.8

Coenzyme transport and metabolism (H)	2.1	-	0.7	-

Lipid transport and metabolism (I)	1.3	-	0.7	-

**Translation, ribosomal structure and biogenesis (J)**	**22.8**	-	1.5	-

Transcription (K)	3.4	-	3.6	-

**Replication, recombination and repair (L)**	4.8	-	**7.2**	0.7

Cell wall, membrane, envelope biogenesis (M)	3.4	-	6.5	4.3

Cell motility (N)	2.1	1.3	-	-

Posttranslational modification, protein turnover, chaperones (O)	6.2	0.7	3.6	1.5

Inorganic ion transport and metabolism (P)	2.8	0.7	2.9	2.9

Secondary metabolites biosynthesis, transport and catabolism (Q)	3.4	0.7	0.7	-

General function prediction only (R)	2.1	-	4.3	2.1

**Function unknown (S)**	4.8	2.1	**39.9**	**15.2**

Signal transduction mechanisms (T)	4.1	1.3	1.5	-

Intracellular trafficking, secretion, and vesicular transport (U)	4.8	-	1.5	1.5

Defense mechanisms (V)	2.1	-	1.5	-

**Without COG (WC)**	5.5	2.1	**8.7**	4.3

Total protein	144	17	143	60

Namely exponential phase (6 h) and the stationary phase (24 h). The relative contribution to each COG of proteins with a potential signal peptide (SP) is indicated. Functional groups with a major abundance in proteins are shown in bold letters and numbers.

The presence of proteins in the groups "production and energy conversion" and "metabolism and transportation of inorganic ions" could be related to the survival of the bacteria to adverse conditions and could contribute to diminish the levels of toxic substances, for instance superoxide dismutase. On the other hand, some proteins like malate dehydrogenase involved in the Krebs cycle, appear in most secretomes previously reported [1,25], suggesting that it could have other physiological role in the bacterium as part of the bacterial secretome.

The presence of superoxide dismutase (SodB) under both studied conditions, and catalase-peroxidase (KatG) in stationary phase, in the *R. etli *CE3 secretome, (Additional file 4), suggest that these proteins could participate in the neutralization of the oxidative burst, by which plants challenge infecting bacteria. SodB is responsible for the inactivation of O_2_^- ^and H_2_O_2 _and is a virulence factor for some phytopathogenic bacteria [26]. In this way, the presence of this protein in the *R. etli *secretome could be crucial for the establishment of the symbiosis, in addition to its remarkable role in the maintenance of the symbiosis as an intracellular protein [26].

The high-molecular-weight heat shock proteins Hsp60 (GroEL) and Hsp70 (DnaK) were also identified, the Hsp60 in both growth phases and Hsp70 only in stationary phase. These proteins may function as chaperons in the type II and type IV secretion systems helping to fold other proteins accumulated in the acidic region, as reported previously in the *E coli *EHEC EDL933 and EPEC E2348/69 extracellular proteomes [27]. For *Legionella pneumophila *and *Helicobacter pylori*, Hsp60 and Hsp70 were reported to be involved in colonization, attachment, and invasion [28]. In addition, previous studies have demonstrated the role of chaperones in secretion systems trough chaperone-usher pathway [29]. Therefore, the presence of these proteins in the *R. etli *extracellular proteome could suggest that they take part in the colonization of the plant.

In stationary phase NodTch protein was identified, this protein was founded to be essential for free-living *R. etli*. The authors proposed that NodTch may play a role in outer membrane stability or in chromosome segregation instead of, or in addition to, drug efflux [30].

We also identified flagellin-like proteins in both growth phases (FlaCch1, FlaCch2, FlaCch3 and FlaCe), and FlgD (flagellar basal-body rod protein) specifically in exponential phase, which are typical components of different bacterial secretomes [31-33]. These proteins are exported *via *a translocation complex involved in the assembly of flagella, which is related to the type III secretion system of Gram-negative bacteria [34]. However, in some cases, the transportation of flagellar proteins should not be considered as secretion in a general sense, because most of proteins to be transported are not intentionally exported into the media, but result from some leaks or spill-over during construction [35].

Two proteins identified as cold shock proteins (CspA4 and CspA5) were found specifically in the secretome of exponential phase. In tobacco cell cultures, it was suggested that cold shock proteins are a novel class of bacterial elicitors for which tobacco and other *Solanaceae *have evolved specific and sensitive chemoperception systems and can potentially serve as MAMPs [36]. Because lysis of bacteria under natural low titre conditions would probably not generate sufficient amounts of Csp to be detected by the plant, an alternative mechanism of protein release other than active secretion can be taken into consideration.

## Bioinformatics analysis of the secreted proteins

The Signal P 3.0 Server program (http://www.cbs.dtu.dk/services/SignalP-2.0/), was used to scan the identified proteins for *N*-terminal signal peptides, indicative for type II and tat-mediated protein transport secretion system [37]. The first 60 amino acids from the *N*-termini were examined by the Signal P software, and only search results with a probability score higher than 0.98 were considered. This analysis revealed that 24.21% of the extracellular proteins identified in this study, (12.95% in the exponential phase and 35.60% in stationary growth phase) had putative secretion signal peptides and were probably secreted via a sec-dependent pathway (Table 1). Which is apparently more active in *R. etli *in the late stages of bacterial growth, in *X. campestri *was previously reported that the 53% of extracellular proteome had signal peptides in early stationary growth phase [1].

Since the software Signal P does not differentiate signal peptides for secretion system type II and Tat-secretion, it was necessary to do a manual search of all signal peptide sequences in order to find a twin arginine motif that is typical of Tat-secretion signal [37]. The twin-arginine motif could only be found in five of the putative secretion signals: Omp1, Omp2, MpA (outer membrane lipoprotein), PstS (phosphate ABC transporter, substrate-binding protein) and FabB (3-oxoacyl-(acyl-carrier-protein) synthase I protein) (data not show). These proteins are the only candidates to traverse the inner membrane in their folded conformation.

Extracellular proteins of *R. etli *with a putative signal peptides, identified in culture supernatant in exponential phase growth, include proteins for transport of carbohydrates (ChvE, AglE, and FrcB) and transport of amino acids (BraC1, BraC2, and DppAch1) (Additional file 1). In general, 30 proteins with signal peptide, identified in both growth phases, could be involved in transport processes. For stationary phase growth, the best represented group of proteins with signal peptides were proteins of unknown function (21 proteins). For some of these, we detected by bioinformatics analysis specific domains for proteases and peptidases (Table 1 and Additional file 2). These classes of enzymes are known to be secreted by pathogenic bacteria [38].

## Conclusion

The results presented here render a more detailed picture of the *R. etli *CE3 extracellular proteome. A total of 384 proteins were identified by LC-MS/MS in two experimental replicates of exponential and stationary phases, and 607 proteins in total, which reveals an abundance of secreted proteins by *R. etli *in both phases of growth and represents the first report about the extracellular proteome in this bacterium. This study also provides a basis for comparative studies of the secretome in different growth conditions for *R. etli*. Our results suggest that the secretome of *R. etli *consists mainly of two groups of proteins. The actively secreted proteins are extracellular enzymes (mostly degradation enzymes) and proteins that bind nutrients and extracellular appendages. The secretion and synthesis of these proteins is apparently regulated by cellular phase growth. The second group includes proteins that have functions in the cytosol and are not actively secreted but may be released into the culture medium due to lysis of bacteria or by other, yet unknown mechanisms.

In addition, in the exponential phase of growth we found a large number of ribosomal proteins. The role that these proteins play in the extracellular proteome remains unknown. Our results also showed many proteins without assigned function mainly, but not exclusively, in stationary phase. Further studies are needed to know the function that these proteins play in the extracellular proteome, and the study of these proteins will clarify aspects of *R. etli *and its interaction with the environment, since the extracellular proteome must be considered an essential component of the bacterial cell, which plays an important role in at least three fundamental processes; protection against stresses, cell survival and colonization of the host.

## Competing interests

The authors declare that they have no competing interests.

## Authors' contributions

NM carried of the experiments and prepared a first draft of the manuscript, GMH supplied methodological expertise and carried of some experiments, SE conceived the study, participated in its design and coordination, and wrote the final manuscript. All authors read and approved the final manuscript.

## Supplementary Material

Additional file 1 **Proteins secreted in exponential phase of growth in *R. etli***. description of all identified proteins in exponential growth phase in *R. etli*. In this table, we show the description of the proteins identified the score, the number of peptides, the molecular weight and isoelectric point. Click here for file

Additional file 2 **Proteins secreted in stationary phase of growth in *R. etli***. description of all identified proteins in stationary growth phase in *R. etli*. In this table, we show the description of the proteins identified the score, the number of peptides, the molecular weight and isoelectric point. Click here for file

Additional file 3 **Comparison of two-dimensional gels in exponential (6 h) andstationary (24 h) growth phase of *R. etli***. Comparison of 2D gels of secreted proteins and cytoplasm proteins in exponential and stationary growth phase of *R. etli*. In order to assess if proteins originated from cell lysis, and hence contaminated the secretome, we compared the protein profiles of whole cell lysates with those of extracellular proteins. Click here for file

Additional file 4 **Common proteins in both phases of growth of *R. etli***. Descriptions of all the common identified proteins are secreted in exponential and stationary growth phase in *R. etli*. In this table, we show the description of the proteins identified the score, the number of peptides, the molecular weight and isoelectric point. Click here for file

## References

[B1] Watt SA Patschkowski T Niehaus K Comprehensive analysis of the extracellular proteins from *Xanthomonas campestris *pv. *campestris *B100 Proteomics 2005 5 153 167 10.1002/pmic.200400905 15619296

[B2] Kambara K Ardissone S Kobayashi H Saad MM Schumpp O Broughton WJ Deakin WJ Rhizobia utilize pathogen-like effector proteins during symbiosis Molecular Microbiology 2009 1 192 106 10.1111/j.1365-2958.2008.06507.x 19019163

[B3] Gohar M Gilois N Graveline R Garreau C Sanchis V Lereclus D A comparative study of *Bacillus cereus*, *Bacillus thuringiensis* and *Bacillus anthracis* extracellular proteomes Proteomics 2005 5 3696 3711 10.1002/pmic.200401225 16167365

[B4] Encarnación S Dunn M Willms K Mora J Fermentative and aerobic metabolism in *Rhizobium etli* J Bacteriol 1995 177 3058 3066 7768801 10.1128/jb.177.11.3058-3066.1995 PMC176993

[B5] Encarnación S Guzmán Y Dunn MF Hernández M del Carmen Vargas M Mora J Proteome analysis of aerobic and fermentative metabolism in *Rhizobium etli *CE3 Proteomics 2003 3 1077 1085 10.1002/pmic.200300427 12833533

[B6] Baker S Campbell JI Stabler R Nguyen HVM To DS Nguyen DV Farrar J Drug resistance in *Chromobacterium violaceu* Genet Mol Res 2004 3 134 147 15100994

[B7] Hyde SC Emsley P Hartshorn MJ Mimmack MM Gileadi U Pearce SR Gallagher MP Gill DR Hubbard RE Higgins CF Structural model of ATP-binding proteins associated with cystic fibrosis, multidrug resistance and bacterial transport Nature 1990 346 6282 362 365 10.1038/346362a0 1973824

[B8] Barriuso-Iglesias M Schluesener D Barreiro C Poetsch A Martín JF Response of the cytoplasmic and membrane proteome of *Corynebacterium glutamicum *ATCC 13032 to pH changes BMC Microbiology 2008 8 225 10.1186/1471-2180-8-225 19091079 PMC2627906

[B9] Kazemi-Pour N Condemine G Hugouvieux-Cotte-Pattat N The secretome of the plant pathogenic bacterium *Erwinia chrysanthemi* Proteomics 2004 4 3177 3186 10.1002/pmic.200300814 15378709

[B10] Watt SA Wilke A Patschkowski T Niehaus K Comprehensive analysis of the extracellular proteins from *Xanthomonas campestris *pv. *campestris *B100 Proteomics 2005 5 153 167 10.1002/pmic.200400905 15619296

[B11] Hansmeier N Chao TC Kalinowski J Pühler A Tauch A Mapping and comprehensive analysis of the extracellular and cell surface proteome of the human pathogen *Corynebacterium diphtheriae* Proteomics 2006 6 2465 2476 10.1002/pmic.200500360 16544277

[B12] Gohar M Gilois N Graveline R Garreau C Sanchis V Lereclus D A comparative study of *Bacillus cereus*, *Bacillus thuringiensis *and *Bacillus anthracis *extracellular proteomes Proteomics 2005 5 3696 3711 10.1002/pmic.200401225 16167365

[B13] Mawuenyega KG Forst CV Dobos KM Belisle JT Chen J Bradbury EM Bradbury AR Chen X *Mycobacterium tuberculosis* functional network analysis by global subcellular protein profiling Mol Biol Cell 2005 16 396 404 10.1091/mbc.E04-04-0329 15525680 PMC539182

[B14] McBroom AJ Kuehn MJ Release of outer membrane vesicles by Gram-negative bacteria is a novel envelope stress response Molecular Microbiology 2007 63 545 558 10.1111/j.1365-2958.2006.05522.x 17163978 PMC1868505

[B15] Zorreguieta A Finnie C Downie JA Extracellular glycanases of *Rhizobium leguminosarum *are activated on the cell surface by an exopolysaccharide-related component Journal of Bacteriology 2000 182 1304 1312 10.1128/JB.182.5.1304-1312.2000 10671451 PMC94416

[B16] Benoıˆt L Manjusha V Tzvi T Vitaly C The VirE3 protein of *Agrobacterium mimics* a host cell function required for plant genetic transformation The EMBO Journal 2005 24 428 437 10.1038/sj.emboj.7600524 15616576 PMC545813

[B17] Aslam SN Erbs G Morrissey KL Newman M-A Chinchilla D Boller T Molinaro A Jackson W Cooper RM Microbe-associated molecular pattern (MAMP) signatures, synergy, size and charge: influences on perception or mobility and host defence responses Molecular Plant Pathology 2009 10 375 387 10.1111/j.1364-3703.2009.00537.x 19400840 PMC6640380

[B18] Gómez-Gómez L Boller T FLS2: an LRR receptor-like kinase involved in the perception of the bacterial elicitor flagellin in *Arabidopsis* Mol Cell 2000 5 1003 1011 10.1016/S1097-2765(00)80265-8 10911994

[B19] Silipo A Molinaro A Sturiale L Dow JM Erbs G Lanzetta R Newman MA Parrilli M The elicitation of plant innate immunity by lipooligosaccharide of *Xanthomonas campestris* J Biol Chem 2005 280 33 660 33 668 16048996 10.1074/jbc.M506254200

[B20] Zipfel C Kunze G Chinchilla D Caniard A Jones JDG Boller T Felix G Perception of the bacterial PAMP EF-Tu by the receptor EFR restricts *Agrobacterium*-mediated transformation Cell 2006 125 749 760 10.1016/j.cell.2006.03.037 16713565

[B21] Soto MJ Domínguez-Ferreras A Pérez-Mendoza D Sanjuán J Olivares J Mutualism versus pathogenesis: the give-and-take in plant-bacteria interactions Cellular Microbiology 2009 11 381 388 10.1111/j.1462-5822.2009.01282.x 19134114

[B22] Song YC Jin S Louie H Ng D Lau R Zhang Y Weerasekera R Al Rashid S Ward LA Der SD FlaC, a protein of *Campylobacter jejuni *TGH9011 (ATCC43431) secreted through the flagellar apparatus, binds epithelial cells and influences cell invasion Mol Microbiol 2004 53 541 553 10.1111/j.1365-2958.2004.04175.x 15228533

[B23] Wlodawer A Li M Gustchina A Oyama H Dunn BM Oda K Structural and enzymatic properties of the sedolisin family of serine-carboxyl peptidases Acta Biochim Pol 2003 50 81 102 12673349

[B24] Okubo A Li M Ashida M Oyama H Gustchina A Oda K Dunn BM Wlodawer A Nakayama T Processing, catalytic activity and crystal structures of kumamolisin-As with an engineered active site FEBS 2006 273 2563 2576 10.1111/j.1742-4658.2006.05266.x 16704427

[B25] Fauvart M Michiels J Rhizobial secreted proteins as determinants of host specificity in the rhizobium-legume symbiosis FEMS Microbiology Letters 2008 1 9 10.1111/j.1574-6968.2008.01254.x 18616593

[B26] Soto MJ Sanjuán J Olivares J Rhizobia and plant-pathogenic bacteria: common infection weapons Microbiology 2006 152 3167 3174 10.1099/mic.0.29112-0 17074888

[B27] Sandkvist M Type II Secretion and Pathogenesis Infection and Inmunity 2001 69 3523 3535 10.1128/IAI.69.6.3523-3535.2001 PMC98326 11349009

[B28] Bumann D Aksu S Wendland M Janek K Zimny-Arndt U Sabarth N Meyer TF Jungblut PR Proteome analysis of secreted proteins of the gastric pathogen *Helicobacter pylori* Infection and Inmunity 2002 70 3396 3403 10.1128/IAI.70.7.3396-3403.2002 PMC128097 12065478

[B29] Waksman G Hultgren SJ Structural biology of the chaperone-usher pathway of pilus biogenesis Nature Reviews Microbiol 2009 7 765 774 10.1038/nrmicro2220 PMC3790644 19820722

[B30] Hernández-Mendoza A Nava N Santana O Abreu-Goodger C Tovar A Quinto C Diminished redundancy of outer membrane factor proteins in rhizobiales: A *nodT *homolog is essential for free-living *Rhizobium etli* Journal Molecular Microbiology Biotechnology 2007 13 22 34 10.1159/000103594 17693710

[B31] Komoriya K Shibano N Higano T Azuma N Yamaguchi S Aizawa SI Flagellar proteins and type III-exported virulence factors are the predominant proteins secreted into the culture media of *Salmonella typhimurium* Molecular Microbiology 1999 34 767 779 10.1046/j.1365-2958.1999.01639.x 10564516

[B32] Süss C Hempel J Zehner S Krause A Patschkowski T Göttfert M Identification of genistein-inducible and Type III-secreted proteins of *Bradyrhizobium japonicum* Journal Biotechnology 2006 126 69 77 10.1016/j.jbiotec.2006.03.037 16707185

[B33] Nielsen H Engelbrecht J Brunak S von Heijne G Identification of prokaryotic and eukaryotic signal peptides and prediction of their cleavage sites Protein Engeneering 1997 10 1 6 10.1093/protein/10.1.1 9051728

[B34] Blocker A Komoriya K Aizawa SI Type III secretion systems and bacterial flagella: Insights into their function from structural similarities Proc Natl Acad Sci USA 2003 100 3027 3030 10.1073/pnas.0535335100 12631703 PMC152238

[B35] Ikeda T Oosawa K Hotani H Self-assembly of the filament capping protein, FliD, of bacterial flagella into an annular structure J Mol Biol 1996 259 679 686 10.1006/jmbi.1996.0349 8683574

[B36] Felix G Boller T Molecular sensing of bacteria in plants. The highly conserved RNA-binding motif RNP-1 of bacterial cold shock proteins is recognized as an elicitor signal in tobacco J Biol Chem 2003 278 8 6201 6208 10.1074/jbc.M209880200 12471032

[B37] Sandkvist M Type II Secretion and Pathogenesis Infection and Immunity 2001 69 3523 3535 10.1128/IAI.69.6.3523-3535.2001 11349009 PMC98326

[B38] Antelmann H Tjalsma H Voigt B Ohlmeier S Bron S van Dijl JM Hecker M A proteomic view on genome-based signal peptide predictions Genome Biology 2001 11 1484 1502 10.1101/gr.182801 11544192

