# Supplementary figures and images for: The extracellular proteome of Rhizobium etli CE3 in exponential and stationary growth phase
Source: Proteome Sci. 2010 Oct 14;8:51. doi: 10.1186/1477-5956-8-51 (PMC2964644; doi:10.1186/1477-5956-8-51)

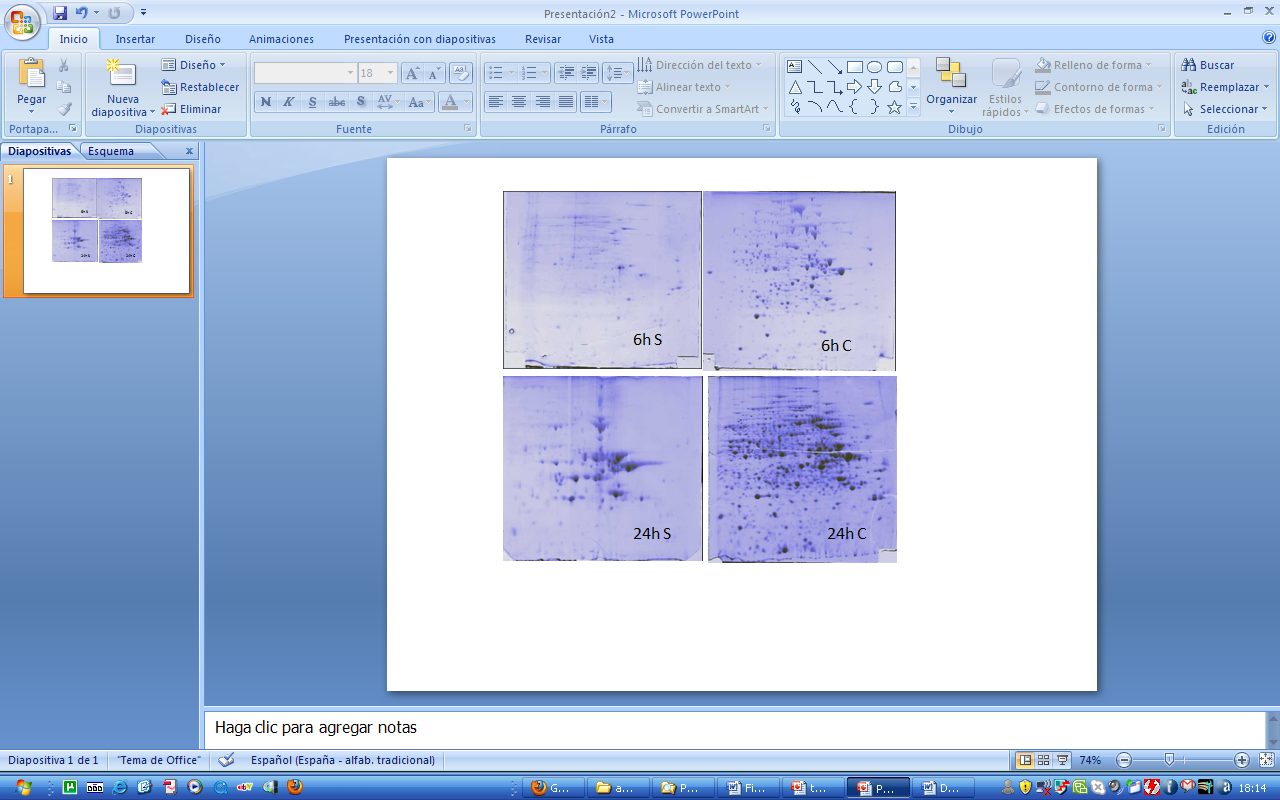

Supplement: Additional file 3 — Comparison of two-dimensional gels in exponential (6 h) andstationary (24 h) growth phase of R. etli. Comparison of 2D gels of secreted proteins and cytoplasm proteins in exponential and stationary growth phase of R. etli. In order to assess if proteins originated from cell lysis, and hence contaminated the secretome, we compared the protein profiles of whole cell lysates with those of extracellular proteins. [file 1477-5956-8-51-S3.DOCX]
